# Supplementary material for: Decarbonization potential of electrifying 50% of U.S. light-duty vehicle sales by 2030
Source: Nat Commun. 2023 Nov 4;14:7077. doi: 10.1038/s41467-023-42893-0 (PMC10625617; doi:10.1038/s41467-023-42893-0)
Supplement: Supplementary file 1 — Supplementary Information [file 41467_2023_42893_MOESM1_ESM.pdf]

Supplementary Information

Decarbonization Potential of Electrifying 50% of U.S. Light-Duty Vehicle Sales by 2030

Maxwell Woody[1], Gregory A Keoleian[1], and Parth Vaishnav[1]

[1]Center for Sustainable Systems, School for Environment and Sustainability, University of Michigan, Ann Arbor, Michigan 48104, USA

13 Pages

8 Figures

3 Tables

Automaker Electrification Goals	Supplementary Table 1	p. S2
Vehicle Sales	Supplementary Figure 1	p. S3
Vehicle Stock	Supplementary Figure 2	p. S4
Vehicle Survival Curves	Supplementary Figure 3	p. S5
Logistic and Linear Growth Comparison	Supplementary Figure 4	p. S5
Vehicle Miles Traveled	Supplementary Figure 5	p. S6
Vehicle Fuel Economies	Supplementary Figure 6	p. S7
Vehicle Production Emissions	Supplementary Figure 7	p. S8
Supplementary Note 1 – CAA 177		p. S9
Supplementary Note 2 – Vehicle Production Emissions		p. S11
Supplementary References		p. S12

Table S1. Electrification and Carbon Reduction Goals of the 10 Largest Automakers by U.S. Sales

Automaker	Electrification Goal(s)	Source
Ford Motor Company	• 50% global sales electric by 2030<br>• All cars and vans zero emissions by 2040<br>• Carbon neutrality by 2050	Integrated Sustainability and Financial Report 2022[1]
General Motors	• 40%-50% EV sales (U.S.) by 2030<br>• All light duty vehicles zero emissions by 2035<br>• Carbon neutrality by 2040	2021 Sustainability Report[2]
Honda Motor Company	• 40% ZEV sales in major markets by 2030<br>• 80% ZEV sales in major markets by 2035<br>• Carbon neutrality by 2040	Sustainability Report 2022[3]
Hyundai Kia Auto Group	• 100% EV sales in Europe by 2035<br>• 100% EV sales in major markets by 2040<br>• Carbon neutrality by 2045	2022 Sustainability Report[4]
Nissan Motor Co	• 100% EV sales in key markets by early 2030s<br>• Carbon neutrality by 2050	Sustainability Report 2022[5]
Stellantis-FCA	• 50% EV sales in U.S. by 2030<br>• 100% EV sales in Europe by 2030<br>• Carbon neutrality by 2038	2021 Corporate Social Responsibility Report[6]
Subaru Corporation	• 40% EV or HEV global sales by 2030<br>• 100% EV or HEV global sales by early 2030s<br>• Carbon neutrality by 2050	Sustainability Report 2022[7]
Tesla	• 100% EV sales	Impact Report 2021[8]
Toyota Motor Corp	• 40% electrified sales by 2025 (North America)<br>• 70% electrified sales by 2030 (North America)<br>• Carbon neutrality by 2050	2021 North American Environmental Report[9]
Volkswagen Auto Group	• 50% EV sales in major markets by 2030<br>• Carbon neutrality by 2050	Sustainability Report 2021[10]

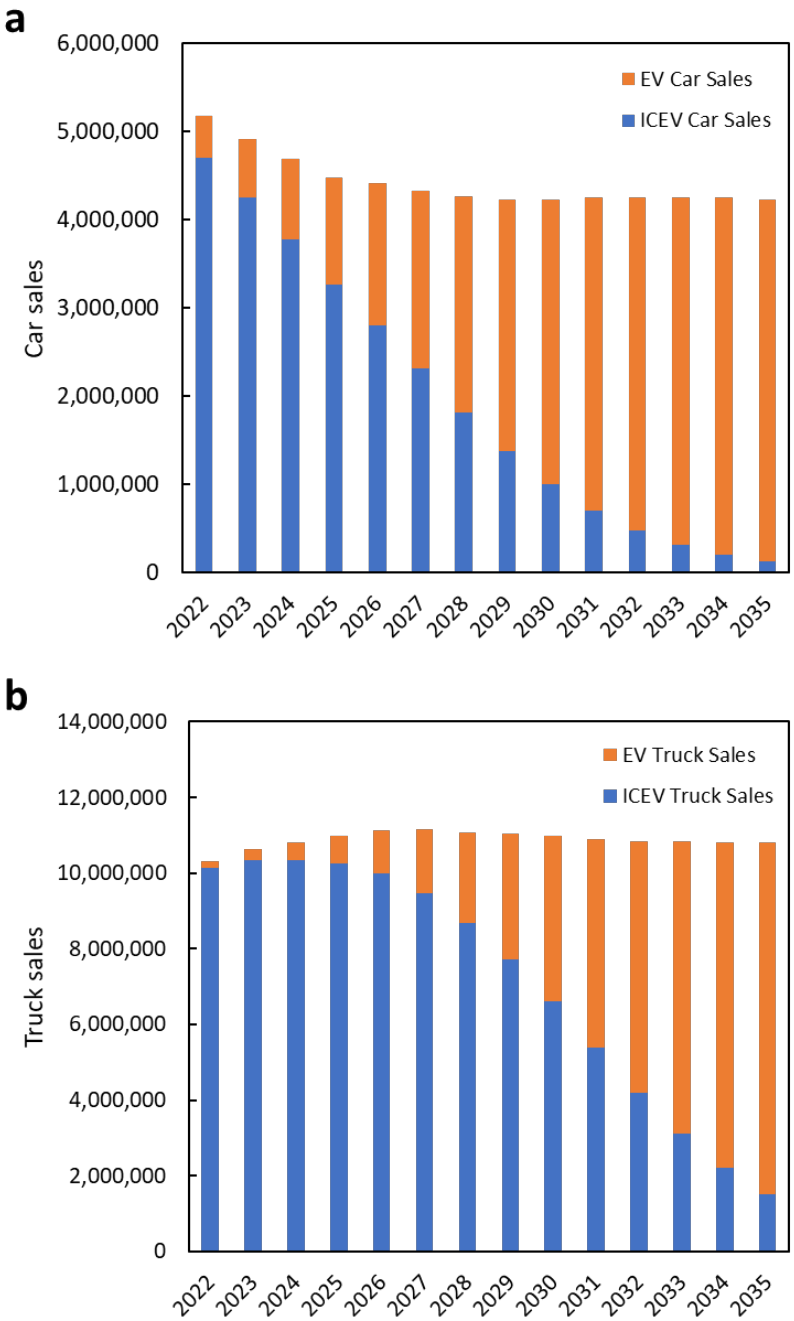

Supplementary Figure 1. Light duty vehicle sales projections

Vehicle sales projections for electric vehicles (orange) and internal combustion engine vehicles (blue) from 2022 to 2035 for a) cars and b) light trucks

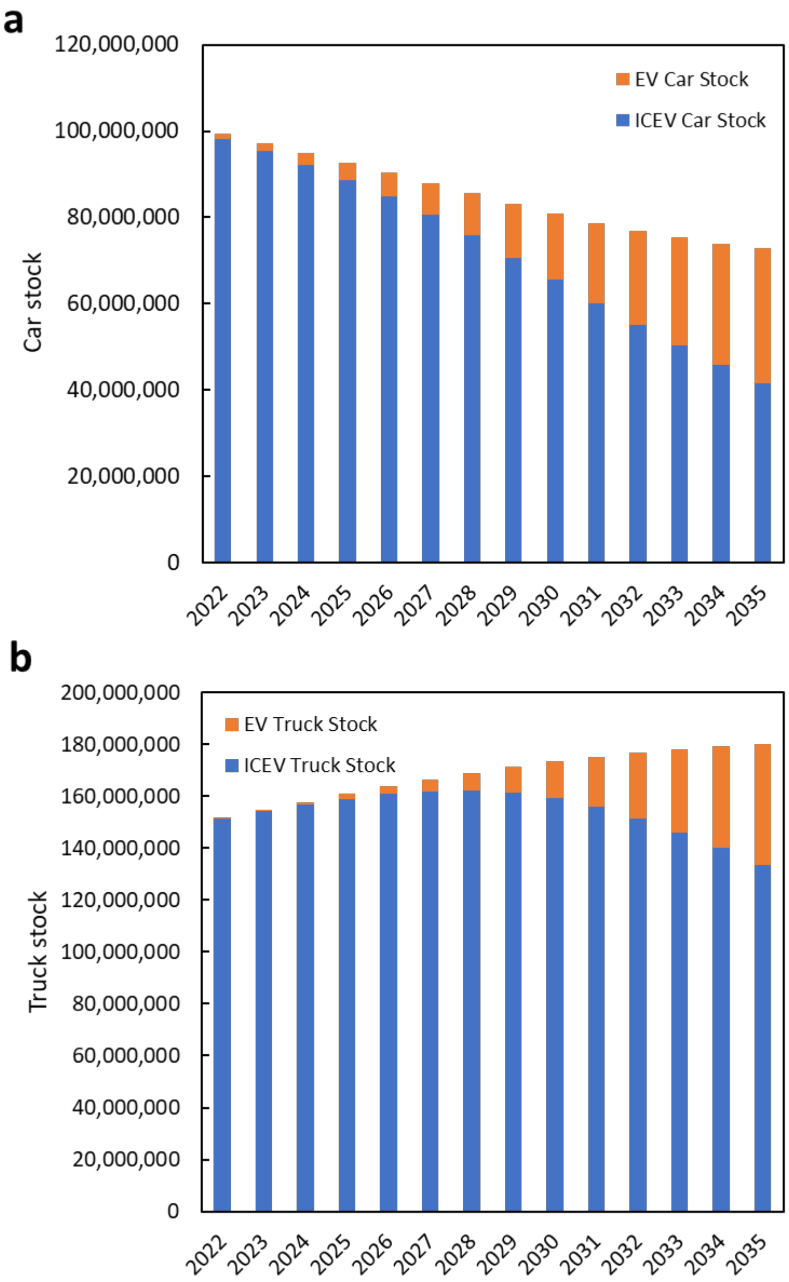

Supplementary Figure 2. Light duty vehicle stock projections

Vehicle stock projections (number of total vehicles on the road) for electric vehicles (orange) and internal combustion engine vehicles (blue) from 2022 to 2035 for a) cars and b) light trucks

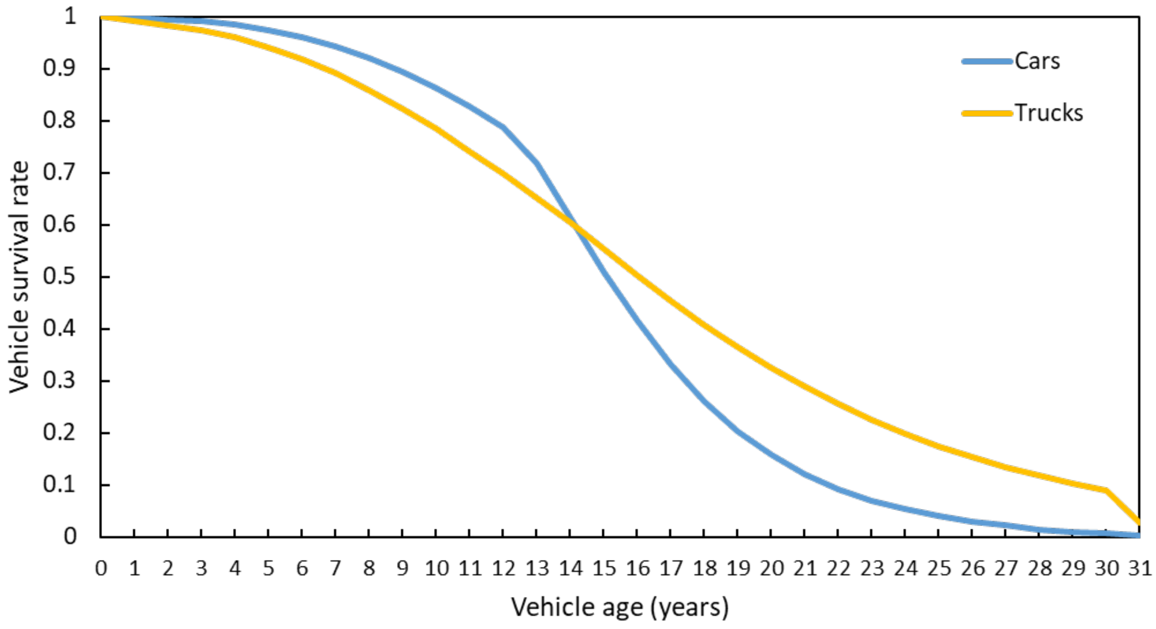

Supplementary Figure 3. Light duty vehicle survival curves

Vehicle survival rate for by vehicle age for cars (blue) and light trucks (yellow) from from U.S. EPA[11]

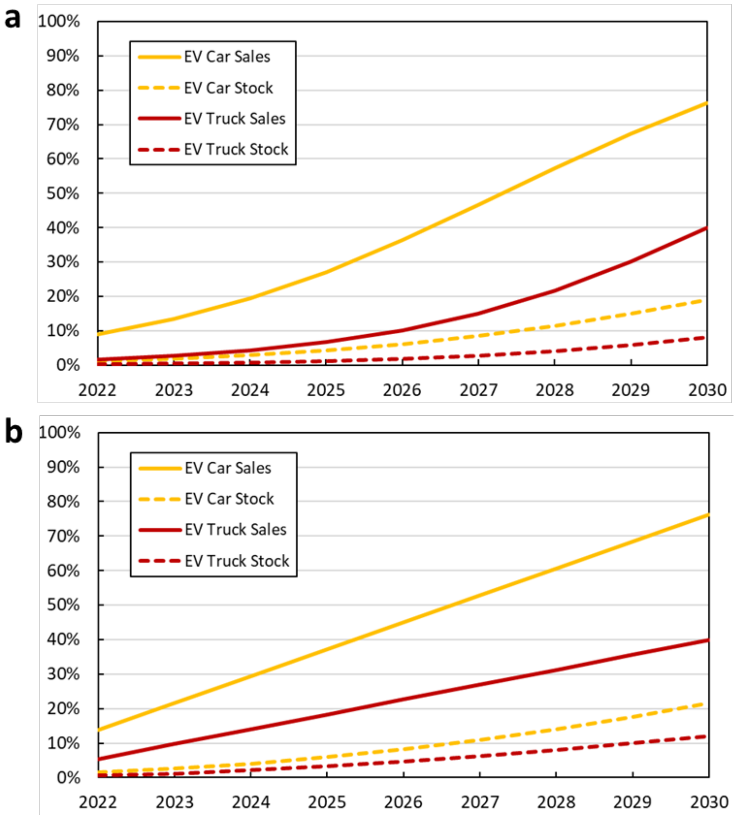

Supplementary Figure 4. Pathways to 50% electric vehicle sales in 2030
Electric car (yellow) and truck (red) sales (solid lines) and stock (dotted lines) from 2022 levels to a 50% sales share in 2030 with a) logistic growth of electric vehicle sales resulting in 11% EV stock in 2030, and b) linear growth of electric vehicle sales resulting in 15% EV stock in 2030.

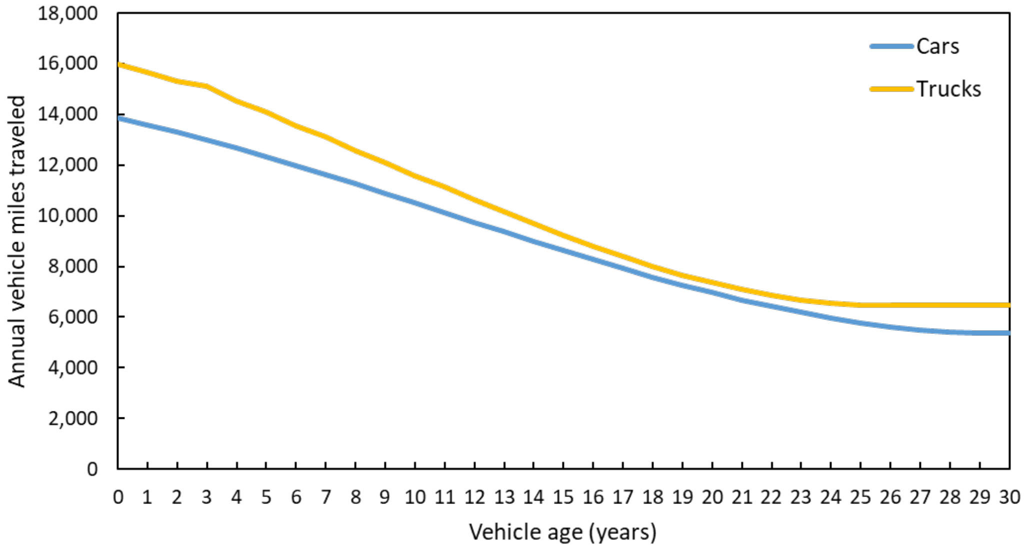

Supplementary Figure 5. Annual vehicle miles traveled by vehicle age
Annual vehicle miles traveled by vehicle age for cars (blue) and trucks (yellow) from the Transportation Energy Data Book[12]

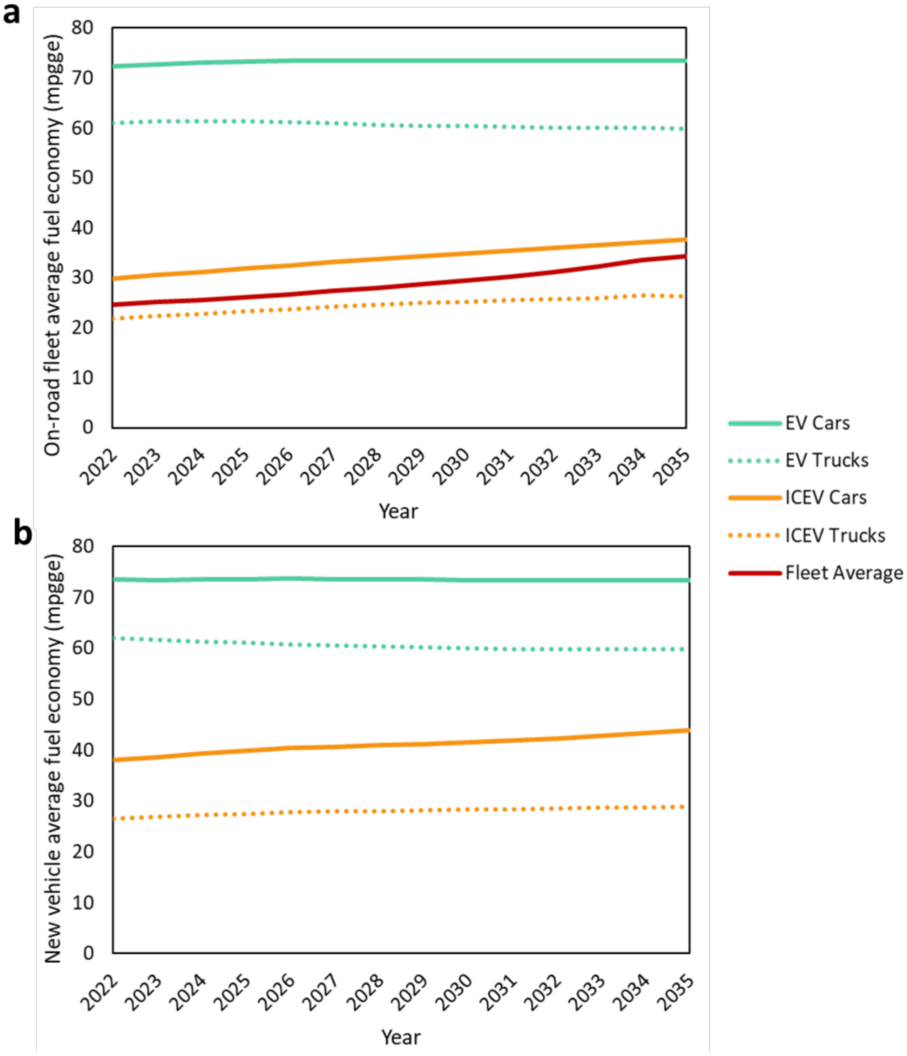

Supplementary Figure 6. Average fuel economy by year

Average fuel economy in miles per gallon of gasoline equivalent for electric vehicles (green) and internal combustion engine vehicles (orange), differentiated by cars (solid lines) and trucks (dotted lines) for a) the on-road vehicle fleet and b) new vehicles sold in that year. The fleetwide average is shown in red. Note that EV fuel economy includes a charger efficiency of 88%.

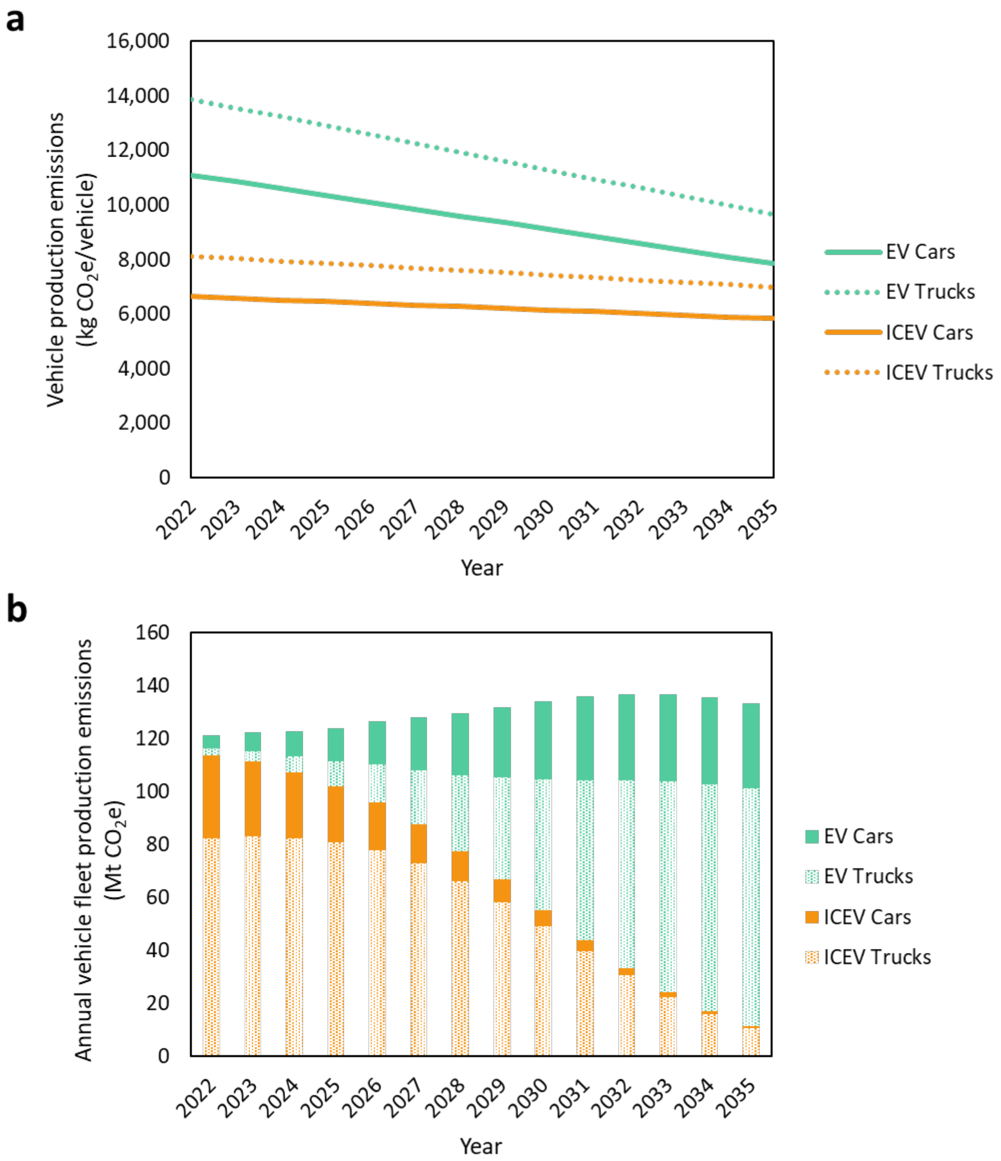

Supplemental Figure 7. **Vehicle production emissions**

Vehicle production emissions electric vehicles (green) and internal combustion engine vehicles (orange) differentiated by cars (solid) and trucks (dashed or shaded), for a) individual new vehicles from 2022-2035 and b) the vehicle fleet in each year from 2022-2035

Supplemental Note 1 – Clean Air Act Section 177 States

In our sensitivity analysis presented in the main text we only consider states that have adopted the ZEV program (Table S2). States that are projected to exceed the goal of the ZEV program in our base scenario were not changed in the sensitivity case (California and Washington). The states that fell short were adjusted so that they met the 67% EV sales in 2030 goal exactly. The difference in sales percentage for states that were adjusted between the base case and the sensitivity case are shown in Fig. S8, and results are compared in Table S3.

Table S2. States that have adopted low emission vehicle (LEV) or zero emission vehicle (ZEV) targets under section 177 of the Clean Air Act[13]

State	LEV Regulations, ZEV program, or Both	Year of ZEV Program Adoption
California	Both	1990
New York	Both	1996
Massachusetts	Both	1995
Vermont	Both	2000
Maine	Both	2001
Pennsylvania	LEV Only	-
Connecticut	Both	2008
Rhode Island	Both	2008
Oregon	Both	2009
New Jersey	Both	2009
Maryland	Both	2011
Delaware	LEV Only	-
Colorado	Both	2023
Minnesota	Both	2025
Nevada	Both	2025
Washington	Both	2025
Virginia	Both	2025
New Mexico	Both	2026
Washington, DC	LEV Only	-

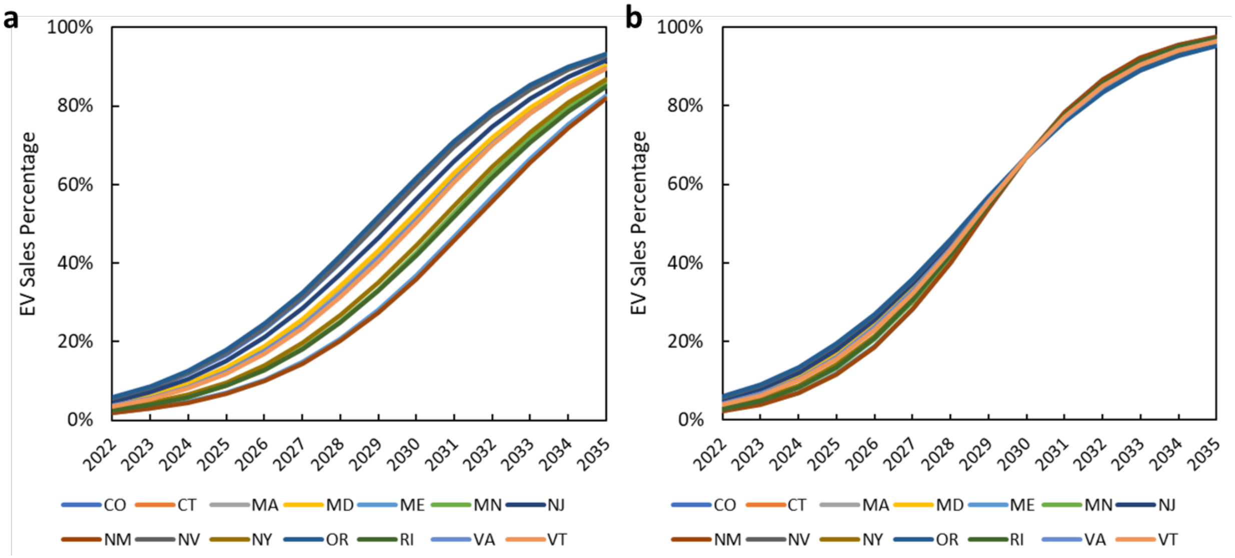

Supplemental Figure 8. Electric vehicle sales percentages in different cases

Electric vehicles sales percentage in the states that are adjusted between a) the base case and b) the CAA section 177 sensitivity case. Sales percentages for all other states are unchanged between the two cases.

Table S3. Comparison of base case and sensitivity case results

	2030		2035	
	Base	Meeting CAA	Base	Meeting CAA
EV Sales (million vehicles)	7.6	8.0	13.4	13.5
EV Sales %	50%	52.3	89.0%	89.9%
EV Stock (million vehicles)	29.3	30.7	80.0	80.2
EV Stock %	11.5%	12.1%	30.8	31.7
Emissions – BAU grid (Megatonnes CO_2e)	1209	1205	1001	993
Emissions – Decarb. grid (Megatonnes CO_2e)	1191	1188	882	872

Supplemental Note 2 – Vehicle Production Emissions

According to the 2021 EPA Automotive Trends Report[14], car sales in the U.S. in 2021 were approximately 70% sedans and 30% “car SUVs”. Truck sales consisted of 75% minivans or “truck SUVs”, and 25% pickup trucks. Woody et al.[15], provides production phase emissions for ICEVs and EVs in three vehicle classes: sedans, SUVs, and pickup trucks. We use the sales ratios from the automotive trends report along with the emissions values reported in Woody et al., to approximate the emissions for the two vehicle categories used in this study (cars and light trucks), as shown in equations S1 and S2.

$$Car\ Emissions = 70\% * Sedan\ Emissions + 30\% * SUV\ Emissions \qquad (S1)$$

$$Light\ Truck\ Emissions = 75\%\ SUV\ Emissions * 25\%\ Pickup\ Emissions \qquad (S2)$$

Where *Sedan Emissions, SUV Emissions,* and *Pickup Emissions* are the production phase emissions for each vehicle powertrain (EV or ICEV) from Woody et al.

Supplemental References

1. Ford Motor Company. *Integrated Sustainability and Financial Report 2022*. https://corporate.ford.com/content/dam/corporate/us/en-us/documents/reports/integrated-sustainability-and-financial-report-2022.pdf (2022).

2. General Motors. *2021 Sustainability Report*. https://www.gmsustainability.com/_pdf/resources-and-downloads/GM_2021_SR.pdf (2022).

3. Honda Motor Co. *Honda Sustainability Report 2022*. https://global.honda/sustainability/cq_img/report/pdf/2022/Honda-SR-2022-en-all.pdf (2022).

4. Hyundai. *Road to Sustainability 2022 Sustainability Report*. https://www.hyundai.com/content/hyundai/ww/data/csr/data/0000000050/attach/english/hmc-2022-sustainability-report-en-v7.pdf (2022).

5. Nissan Motor Corporation. *Sustainability Report 2022*. https://www.nissan-global.com/EN/SUSTAINABILITY/LIBRARY/SR/2022/ASSETS/PDF/SR22_E_All.pdf (2022).

6. Stellantis. *2021 Corporate Social Responsibility Report*. https://www.stellantis.com/content/dam/stellantis-corporate/sustainability/csr-disclosure/stellantis/2021/Stellantis_2021_CSR_Report.pdf (2022).

7. Subaru. *Sustainability Website 2022*. https://www.subaru.co.jp/en/csr/report/pdf/2022/subaru_sustainability_report2022_all.pdf (2022).

8. Tesla. *Impact Report 2021*. https://www.tesla.com/ns_videos/2021-tesla-impact-report.pdf (2022).

9. Toyota. *Toyota North American Environmental Report*. https://www.toyota.com/content/dam/tusa/environmentreport/downloads/2021_Toyota_NAER.pdf (2021).

10. Volkswagen AG. *Sustainability Report 2021*. https://www.volkswagenag.com/presence/nachhaltigkeit/documents/sustainability-report/2021/Nonfinancial_Report_2021_e.pdf (2022).

11. Office of Transportation and Air Quality, U. S. E. P. A. *Proposed Determination on the Appropriateness of the Model Year 2022-2025 Light-duty Vehicle Greenhouse Gas Emissions Standards under the Midterm Evaluation: Technical Support Document (EPA-420-R-16-021)*. https://nepis.epa.gov/Exe/ZyPDF.cgi?Dockey=P100Q3L4.pdf (2016).

12. Davis, S. C. & Boundy, R. G. *Transportation Energy Data Book: Edition 40*. (2022).

13. California Air Resources Board. States that have Adopted California's Vehicle Standards under Section 177 of the Federal Clean Air Act. https://ww2.arb.ca.gov/resources/documents/states-have-adopted-californias-vehicle-standards-under-section-177-federal (2022).

14. US EPA, Office of Transportation and Air Quality, C. D. *The 2021 EPA Automotive Trends Report: Greenhouse Gas Emissions, Fuel Economy, and Technology since 1975*. https://www.epa.gov/automotive-trends/download-automotive-trends-report (2021).

15. Woody, M. *et al.* The role of pickup truck electrification in the decarbonization of light-duty vehicles. *Environmental Research Letters* **17**, 034031 (2022).
